# The impact of translocations on neutral and functional genetic diversity within and among populations of the Seychelles warbler

**DOI:** 10.1111/mec.12740

**Published:** 2014-04-18

**Authors:** David J Wright, Lewis G Spurgin, Nigel J Collar, Jan Komdeur, Terry Burke, David S Richardson

**Affiliations:** * School of Biological Sciences, University of East AngliaNorwich Research Park, Norwich, NR4 7TJ, UK; † NERC Biomolecular Analysis Facility, Department of Animal and Plant Sciences, University of SheffieldSheffield, S10 2TN, UK; ‡ Behavioural Ecology and Self-organization group, Centre for Ecological and Evolutionary Studies, University of GroningenPO Box 11103, Groningen, 9700 CC, The Netherlands; § BirdLife InternationalWellbrook Court, Girton Road, Cambridge, CB3 0NA, UK; ¶ Nature SeychellesRoche Caiman, PO Box 1310, Mahé, Seychelles

**Keywords:** conservation, differentiation, drift, genetic capture, genetic diversity, major histocompatibility complex, re-introduction

## Abstract

Translocations are an increasingly common tool in conservation. The maintenance of genetic diversity through translocation is critical for both the short- and long-term persistence of populations and species. However, the relative spatio-temporal impacts of translocations on neutral and functional genetic diversity, and how this affects genetic structure among the conserved populations overall, have received little investigation. We compared the impact of translocating different numbers of founders on both microsatellite and major histocompatibility complex (MHC) class I diversity over a 23-year period in the Seychelles warbler (*Acrocephalus sechellensis*). We found low and stable microsatellite and MHC diversity in the source population and evidence for only a limited loss of either type of diversity in the four new populations. However, we found evidence of significant, but low to moderate, genetic differentiation between populations, with those populations established with fewer founders clustering separately. Stochastic genetic capture (as opposed to subsequent drift) was the main determinant of translocated population diversity. Furthermore, a strong correlation between microsatellite and MHC differentiation suggested that neutral processes outweighed selection in shaping MHC diversity in the new populations. These data provide important insights into how to optimize the use of translocation as a conservation tool.

## Introduction

The translocation of populations for species conservation and ecosystem restoration is an increasingly common conservation tool (Moritz 1999; Ewen *et al.* 2012; IUCN/SSC 2013). However, translocation success rates have been poor across many taxa, often for unknown reasons (Griffith *et al.* 1989; Wolf *et al.* 1996; Godefroid *et al.* 2011). Close monitoring of translocated populations is crucial if we are to understand the drivers of success and failure (Miller *et al.* 1999; Allendorf & Luikart 2007), but until recently such monitoring has often been absent or inadequate (Fischer & Lindenmayer 2000; Armstrong & Seddon 2008). Translocations have been particularly widely used in the conservation of oceanic island species. Such islands possess some of the most globally threatened and evolutionarily distinct taxa (Kier *et al.* 2009; Lee & Jetz 2011), contributing disproportionally to global biodiversity (Whittaker 1998). Further, the small size of many oceanic islands means that eradication of alien predators and restoration of native biota are achievable goals (Nogales *et al.* 2004; Howald *et al.* 2007), enhancing the prospects of successful translocations.

Many biotic and abiotic factors can influence translocation success, including genetic diversity (Sarrazin & Barbault 1996; Wolf *et al.* 1998; Seddon *et al.* 2007; Groombridge *et al.* 2012). Maintaining genetic diversity is one of the IUCN's three conservation priorities, and its role in species extinction risk is now largely accepted (Spielman *et al.* 2004; Frankham 2005; O'Grady *et al.* 2006). However, translocations typically involve small source populations and limited numbers of founders, resulting in founder effects (e.g. Taylor & Jamieson 2008; Cardoso *et al.* 2009). Furthermore, genetic drift is stronger in smaller populations, will erode genetic diversity (Kimura 1983) and will cause interpopulation divergence if populations are isolated (e.g. Brekke *et al.* 2011). Small, isolated populations also suffer inbreeding (Frankham *et al.* 2002) and inbreeding depression (Charlesworth & Charlesworth 1987). Purging of the mutational load may alleviate this over time (Crnokrak & Barrett 2002), but its effectiveness in wild populations is uncertain (Boakes *et al.* 2007). In the longer term, the loss of genetic diversity also reduces a population's ability to adapt to future challenges, that is its evolutionary potential (Franklin 1980; Franklin & Frankham 1998). Translocations can, therefore, have considerable long-lasting genetic impacts on populations and hence on entire species (Biebach & Keller 2009), and there is a clear need to integrate genetic considerations into translocation programmes (Jamieson & Lacy 2012).

Most studies on the genetic impacts of translocation investigate diversity at putatively neutral markers such as microsatellites (e.g. Larson *et al.* 2002; Taylor & Jamieson 2008; Brekke *et al.* 2011). Such variation may not correlate well with the genetic diversity important to the evolutionary potential of the new populations (Reed & Frankham 2001; Aguilar *et al.* 2004). It may be more relevant to directly assess variation at important functional loci, such as the major histocompatibility complex (MHC). These highly polymorphic loci encode molecules central to antigen recognition in the adaptive immune response of vertebrates (Hughes & Yeager 1998). This key role has made the MHC an attractive candidate for studying functional diversity in and among populations (reviewed in Bernatchez & Landry 2003; Sommer 2005; Spurgin & Richardson 2010). A few studies have compared the impact of translocation on neutral and functional diversity in wild populations (see Sutton *et al.* 2011; Bauer *et al.* 2013; Monzón-Argüello *et al*. 2013). Perhaps the best example to date is a study by Miller and Lambert (2004) which showed that drift outweighed selection in shaping MHC diversity in re-introduced populations of two bird species in New Zealand. However, the reasons for differences in loss of neutral and functional variation during the process of translocation itself remain unclear.

How many individuals to translocate is a difficult question to answer, although it is a central concern for conservationists. Guidelines recommend that ‘adequate’ numbers of founders should be taken (IUCN/SSC 2013), although this is rarely quantified (Tracy *et al.* 2011). The goal of translocation is to establish a ‘viable’ population (IUCN/SSC 2013), often defined by the ‘minimum viable population’ concept, with estimates ranging from 50 individuals for short-term persistence up to 5000 individuals to maintain evolutionary potential (Franklin 1980; Lande 1995; Franklin & Frankham 1998; Willi *et al.* 2006). However, translocations often use <50 founders owing to ecological, logistic and economic constraints (Komdeur 1994; Cardoso *et al.* 2009; Jamieson 2011; Tracy *et al.* 2011). Genetic data have only recently been incorporated into estimates of what constitutes ‘adequate’ founder sizes, even though the genetic consequences of small population size are directly relevant. Weeks *et al*. (2011) introduced the concept of ‘genetic capture’, where the minimum number of individuals to translocate is determined by the capture of ≥95% of source population genetic diversity. Models have also been developed to predict allele retention accounting for post-translocation parameters such as survival, population growth, carrying capacity, overlapping generations and/or specific mating systems (Brekke *et al.* 2011; Tracy *et al.* 2011; Weiser *et al.* 2012). Modelling approaches provide vital benchmarks for conservationists, but there are drawbacks. Importantly, these methods are largely based on the loss of neutral variation in the founding population. Drift may disproportionately reduce variation at functional loci if they are highly variable due to a history of balancing selection (Sutton *et al.* 2011). Therefore, such methods are only useful for functional diversity if the loci are selectively neutral at small population sizes (Tracy *et al.* 2011). Complementing modelling approaches with replicated empirical data on neutral and functional variation, in systems with adequate pre- and post-translocation sampling, should help inform best practice for future translocation programmes.

Here, we present data from a study spanning 23 years and involving four translocations of different founder sizes of the Seychelles warbler (*Acrocephalus sechellensis*), an isolated island species. The objective was to compare the spatio-temporal impact of translocation on neutral and functional diversity within and across populations. First, we characterize neutral and functional genetic diversity in the source population and how that has changed over time. Second, we compare predicted vs. observed levels of genetic capture across different founder sizes. Third, we quantify genetic diversity in the four new translocated populations, including how that compares with variation in the source population and if it changes over time. Fourth, we quantify levels of genetic differentiation among all the Seychelles warbler populations now in existence. Fifth, we estimate the effective population size for each population. We discuss the implications of our findings for the use of translocation in the conservation of this and other endangered species.

## Materials and methods

### Study populations

By the mid-20th century, the Seychelles warbler was on the verge of extinction due to habitat destruction and the introduction of invasive predators, with the last population of 26–50 individuals (Crook 1960; Spurgin *et al*. in review) existing on Cousin Island (4°20′S, 55°40′E, 0.29 km²). This population has been under intense study since 1986 (>96% individuals ringed since 1997, Richardson *et al.* 2001) as part of a long-term project (Komdeur 1992; Richardson *et al.* 2007; Barrett *et al.* 2013). Each year, birds are caught and unringed individuals are identified with a unique combination of coloured leg rings (herein referred to as catch-year samples). A *ca*. 25 μl blood sample is taken from every bird and stored in absolute ethanol. Four translocations have been undertaken as part of the species conservation plan (Richardson 2001), with Cousin as the source for each (Fig. S1, Supporting information). In brief, 29 birds were translocated to both Aride (4°12′S, 55°40′E, 0.68 km²) and Cousine (4°21′S, 55°39′E, 0.25 km²) in 1988 and 1990, respectively (Komdeur 1994). In 2004, 58 birds were translocated to Denis (3°48′S, 55°40′E, 1.42 km², Richardson *et al.* 2006) and in 2011, 59 birds to Frégate (4°35′S, 55°56′E, 2.19 km², Wright & Richardson 2012). For each translocation, founders were selected from across the whole source population based on body condition, age (avoiding very young or old birds), breeding experience and sex, to maximize the chances of population establishment. The translocations were undertaken blind in regard to genetic characteristics, although translocating known first-order relatives was avoided. Movement between populations is virtually absent, with rates of inter-island dispersal at only 0.1% (Komdeur *et al.* 2004). Complete sampling of the founding populations was conducted for Denis and Frégate, but the Aride and Cousine translocations were undertaken prior to routine blood sampling and so few founders were sampled (4 of 29 in each translocation). The following catch-year samples were used in this study: Cousin 1993, 2005, 2011; Aride 1993, 2005, 2011; Cousine 1997, 2005, 2011; Denis 2004, 2011; Frégate; 2011 (sample sizes in Table1).

**Table 1:** Catch-year sampling regime and marker summary statistics for microsatellite and major histocompatibility complex (MHC) data across the five Seychelles warbler populations. Cousin is the source of all other populations. Abbreviations are number of individuals scored (N), expected heterozygosity (H_E_), total number of MHC alleles in the population (Alleles), mean MHC alleles per individual (MHC/ind), nucleotide diversity (Pi) and an index of allelic richness (Theta K)

Island	Catch year	Microsatellites			MHC				
		N	H_E_	Allelic richness	N	Alleles	MHC/ind	Pi	Theta K
Cousin	1993	49	0.49	2.96	52	10	3.94	19.76	2.05
	2005	169	0.48	2.89	156	10	4.61	19.71	1.54
	2011	163	0.48	2.91	91	10	4.71	19.50	1.71
Aride	1993	27	0.49	2.82	27	10	4.85	18.84	2.34
	2005	30	0.47	2.84	30	10	4.67	18.68	2.30
	2011	29	0.45	2.81	29	10	4.93	18.97	2.28
Cousine	1997	24	0.45	2.60	23	10	5.09	19.70	2.43
	2005	29	0.42	2.59	29	9	4.03	19.13	2.10
	2011	30	0.45	2.70	30	10	4.17	20.16	2.05
Denis	2004	58	0.50	2.95	56	10	4.77	19.69	1.91
	2011	35	0.48	2.85	30	10	4.97	19.25	2.25
Frégate	2011	59	0.49	2.90	58	10	4.55	19.52	1.92

### Molecular and statistical analyses

Statistical analyses were performed using r version 2.15 (R Development Core Team 2012), unless stated. Samples were genotyped at 30 microsatellite loci following Spurgin *et al*. (in review). Rare alleles (frequency <0.01) were verified by two or more independent PCRs from different samples. We tested for deviations from Hardy–Weinberg equilibrium and linkage disequilibrium between loci using genepop version 4.1 (Raymond & Rousset 1995b) and for null alleles using cervus version 3.0 (Marshall *et al.* 1998). Genetic diversity in each catch-year sample on each island was quantified by calculating expected heterozygosity (H_E_) using arlequin version 3.5 (Excoffier & Lischer 2010). Allelic richness was quantified using a rarefaction approach in hp-rare version 1 (Kalinowski 2005) as sample size differences can bias the estimations (Leberg 2002).

Variation at exon 3 of MHC class I, which codes for the peptide-binding region involved in antigen recognition (Hughes & Yeager 1998), was screened using reference strand-mediated conformation analysis (RSCA) using the primers from Richardson & Westerdahl (2003), following the method of Worley *et al*. (2008). Each segregating RSCA variant corresponded to a unique 255-bp amino acid coding sequence (hereafter termed ‘allele’ for simplicity, Richardson & Westerdahl 2003). Ten MHC class I alleles have been detected in the Seychelles warbler, with individuals possessing 2–8 alleles each, suggesting that at least four class I loci are amplified (Richardson & Westerdahl 2003). Our primers were sited within exon 3. Consequently, we were not able to screen all the variation within this exon, and it is possible that some additional polymorphism exists (e.g. Llaurens *et al.* 2012). However, the amplicon includes all the codons of the peptide-binding region where we expect most variation to be found (Hughes & Yeager 1998). Further, to minimize the effect of this issue, we employed two primer sets which vary at the 3′ end where a known polymorphism occurs (see Richardson & Westerdahl 2003). Finally, any missed variation would not affect the main results or conclusions of the present study which seeks to address how the variation we have screened is captured across translocated populations. It is impossible at present to identify locus zygosity, due to homogeneity of alleles between multiple, duplicated loci within the MHC (Westerdahl 2007). Instead, we measured MHC diversity by calculating the total number of different alleles in each catch-year sample, the mean number of alleles per individual (MHC/ind), nucleotide diversity (Pi) and theta *K* (allelic richness) in arlequin by entering the nucleotide sequence and number of individuals carrying each allele as haplotype data, following Miller *et al*. (2010). This approach may overestimate rare alleles and underestimate common ones but is the best available (Ekblom *et al.* 2007).

Changes in genetic diversity over time were analysed using a randomization approach (Manly 1997). For each diversity measure, the data from the earliest and latest years of each population were pooled and randomly resampled with replacement 100 000 times. The *P* value was calculated as the proportion of times the difference between the means of the resampled data sets were equal to or greater than the observed difference between earliest and latest years. Differences in microsatellite variation between years were also tested using a global differentiation exact test (Raymond & Rousset 1995a) in arlequin, with a 30 000 step Markov chain. Seychelles warblers can live up to 17 years (Brouwer *et al.* 2010) and are routinely sampled multiple times throughout their life as part of this long-term study system (e.g. Barrett *et al.* 2013). To check for any effect of including the same individuals across catch-year samples, diversity measures were also compared for lay-year (year of hatching, estimated at first ringing) cohorts on Cousin. Patterns of diversity across lay years and catch years were qualitatively the same (data not shown) so only catch-year data are reported. This enabled us to use the largest sample sizes available and hence most accurate estimations of diversity for each year in our analyses. Differences in diversity between populations were assessed using the same randomization approach. As we observed no differences in variation within islands over time (see Study populations), for between-island comparisons, samples were pooled for each island to improve the accuracy of the rarefaction measures (i.e. allelic richness). Differences in mean MHC/ind between islands were tested using a Kruskal–Wallis test.

To model the expected genetic capture at each translocation, we constructed rarefaction curves of observed allelic richness using the 2011 Cousin sample (microsatellites; *n* = 163, MHC; *n* = 91) with 1000 repetitions. As we did not have source population diversity data prior to every translocation and diversity did not change over time within islands (see Study populations), we used the 2011 sample as a proxy for pretranslocation diversity at all four genetic capture events. A proportionally greater loss of genetic diversity is expected from microsatellite loci of higher initial diversity (Hedrick 1999), so microsatellite genetic capture was investigated using two models: all loci (*n* = 30) and ‘diverse loci’ only (*n* = 7), defined as possessing ≥4 alleles, a threshold that separates out the *ca*. 25% most polymorphic loci in our data set. As mean MHC/ind varied over time (see Study populations), a second MHC curve was constructed using the 1993 Cousin sample (*n* = 52) to check the accuracy of capture estimates for the earlier translocations to Aride (1988) and Cousine (1990). The rarefaction curves were then used to estimate the expected allelic richness captured during the translocations by taking the mean of the 1000 rarefaction repetitions for each number of translocated birds (Aride/Cousine 29, Denis 58, Frégate 59). To test model accuracy, we compared the observed microsatellite allelic richness captured for Denis and Frégate (where we had complete founder samples) with the distribution of expected values generated by rarefaction. A one-tailed *P* value was obtained by calculating the percentile of simulated data in which the observed mean was located.

Population structure within and among islands was tested by calculating microsatellite and MHC pairwise *F*_ST_ in arlequin across all twelve catch-year samples. We also calculated pairwise D_EST_ (Jost 2008), using the r package MMOD (Winter 2012) for microsatellites and SPADE (Chao & Shen 2010) for MHC. The measures were strongly correlated for both microsatellites and MHC (both *r*_M_ > 0.90, *P *<* *0.001); hence, only *F*_ST_ is reported. The relationship between pairwise microsatellite and MHC *F*_ST_ values was assessed using a Mantel test. A Bayesian algorithm was also implemented in structure version 2.3 (Pritchard *et al.* 2000) to determine the most likely number of genetic clusters (*K*). As all islands have recent common ancestry, limited differentiation was expected, so samples from the latest sampling period (2011) were used along with a model of no admixture, prior information on sampling location and correlated allele frequencies, features which are suited to detecting subtle structure (Hubisz *et al.* 2009). We carried out four independent iterations of 500 000 repetitions with a burn-in of 20 000 at each clustering level for *K* = 1–5. We analysed the results using structure harvester (Earl & vonHoldt 2012), which implements the ad hoc ΔK test (Evanno *et al.* 2005), a more accurate estimate of the most likely number of clusters than assessing log probability alone. The structure results were visualized using distruct version 1.1 (Rosenberg 2004).

Estimates of the effective population size (N_e_) of each population were obtained using two different methods: (i) We used the approximate Bayesian computation approach implemented in ONeSAMP (Tallmon *et al.* 2008), with N_e_ priors of 2–150 for each island. This method bases N_e_ on a single population sample (from 2011); (ii) We used a linkage disequilibrium approach in LDNE (Waples & Do 2008) with a random mating model to account for complex patterns of breeding in this species (Komdeur 1992; Richardson *et al.* 2001). The lowest allele frequency allowed was 0.02 to address the trade-off between estimate precision and bias (Waples & Do 2008), although bias should be minimal given the comparatively low polymorphism observed in the microsatellites.

## Results

Microsatellite genotypes were compiled for 658 individuals and MHC genotypes for 581 individuals across the five populations. All populations were in Hardy–Weinberg equilibrium at all microsatellite loci. Linkage disequilibrium was detected in nine different loci pairs across the five islands after sequential Bonferroni correction (26 of 4933 pairwise comparisons significant). However, inconsistency of patterns between and within populations suggests they are not truly linked. Null allele frequency estimates were also inconsistent across years within each island, with no locus frequency (*F*) ≥0.1 more than twice except for locus *Ase3* (F ≥ 0.1 in four catch-year samples, Table S1, Supporting information). Excluding the *Ase3* locus did not qualitatively alter the results (data not shown). All 30 loci were retained in the final analyses.

Microsatellite diversity in the source population on Cousin had an overall mean (± SE) *H*_E_ of 0.49 ± 0.005 and allelic richness of 2.92 ± 0.01. Neither measure differed across years (randomization tests, *H*_E_: *P *=* *0.67; allelic richness: *P *=* *0.85) and the global exact test found no overall differentiation across years (*P *>* *0.99). Mean MHC/ind increased from 1993 (3.93 ± 0.19) to 2011 (4.71 ± 0.16, *P *=* *0.003). Full diversity estimates are given in Table1.

**Figure 1:**
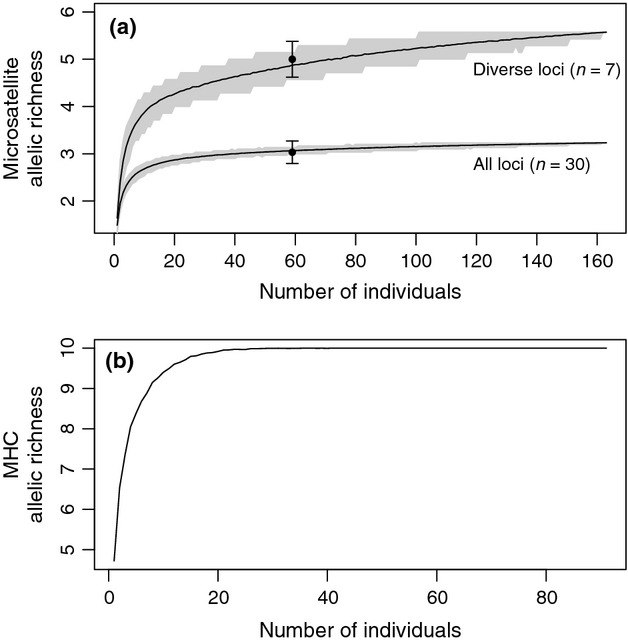
Rarefaction curves constructed from 1000 replicates of the genetic variation observed on Cousin Island in 2011. (a) Microsatellite allelic richness (*n* = 163). Diverse loci have ≥4 alleles, and the shaded areas lie between the 5–95th percentiles. Observed mean allelic richness of Frégate founders (*n* = 59) given as points with SE bars, (b) major histocompatibility complex variation (*n* = 91).

Resampling from the source population, the genetic capture model estimated that 95% (3.07 ± 0.01) of the overall microsatellite allelic richness would be captured with the translocation of 59 individuals, but only 87% (4.86 ± 0.01) of allelic richness for diverse loci. Virtually identical values were observed for 58 individuals (all loci: 3.06 ± 0.01, diverse loci: 4.86 ± 0.01). There was no difference between expected and observed allelic richness translocated to Frégate (all loci: *P *=* *0.36, diverse loci: *P *=* *0.37, Fig.1a) or Denis (all loci: *P *=* *0.35, diverse loci: *P *=* *0.35), indicating a good fit of the genetic capture models. It is therefore reasonable to assume that the rarefaction curve estimates of genetic capture for 29 individuals (all loci = 91%, 2.94 ± 0.10; diverse loci = 85%, 4.45 ± 0.10) provide an accurate representation of genetic diversity initially captured from the source population during the translocations to Cousine and Aride. Across all 30 loci, we observed a loss of eight and ten alleles in the Denis and Frégate translocations, respectively. On Denis, all losses were of rare alleles (frequency < 0.01 in source population) from diverse loci, and on Frégate, two alleles were also lost from less diverse loci, including the biallelic *Pte24*-CEST locus, leading to fixation of a single allele in this population. The lack of founder genotypes or complete population sampling for Aride and Cousine means we could not directly determine the exact roles of genetic capture vs. subsequent drift in allele loss for these two translocations.

The MHC rarefaction curves estimated that *ca*. 20 (2011 sample) to *ca*. 25 (1993 sample) individuals would be required for complete sampling of known class I variation (Fig.1b). All MHC class I alleles were subsequently found in the Denis and Frégate founders at translocation, and in Aride and Cousine in subsequent catch-year samples (Table1).

**Figure 2:**
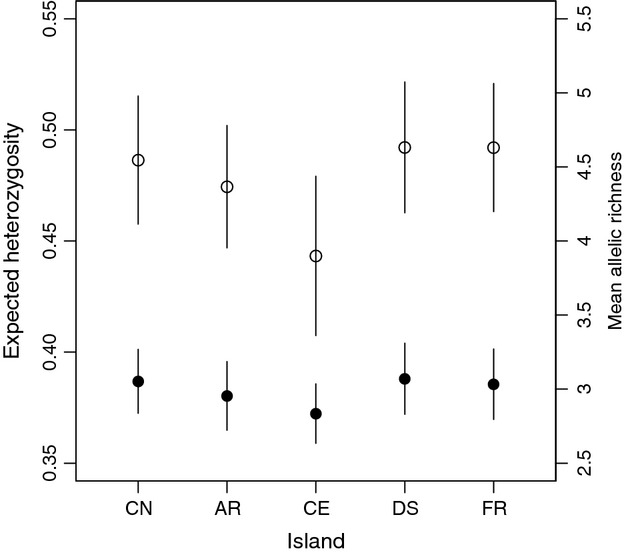
Genetic diversity and differentiation between populations of the Seychelles warbler; H_E_ (white points) and rarefied allelic richness (black points) across all microsatellite loci. Error bars given are SE. CN = Cousin, AR = Aride, CE = Cousine, DS = Denis, FR = Frégate.

Diversity estimates for translocated populations are given in Table1. Although some microsatellite alleles were lost in the translocation process, no significant differences in either microsatellite H_E_ or allelic richness across either the full suite of loci or diverse loci alone were detected between islands (pooled samples, all *P *>* *0.20, Fig.2). There were also no differences in microsatellite diversity over time within each population (all *P *>* *0.30). There was no difference in mean MHC/ind (H = 6.03, *P *=* *0.20) between islands (pooled samples). Similarly, there were no differences in MHC/ind across time within each translocated population (all *P *>* *0.07).

**Figure 3:**
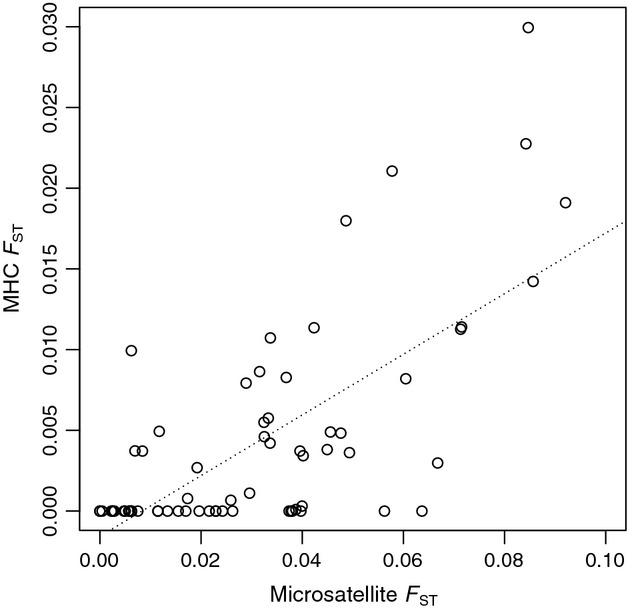
Comparison of microsatellite and major histocompatibility complex *F*_ST_ differentiation across catch-year samples between populations of the Seychelles warbler. Mantel test *r* = 0.69, *P *<* *0.001 (using all island and catch-year pairwise comparisons).

**Figure 4:**
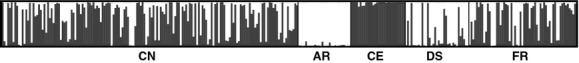
STRUCTURE plot of genetic clustering across the five Seychelles warbler populations in 2011; Cousin (CN), *n* = 163. Aride, *n* = 29. CE, *n* = 30. Denis, *n* = 35. Frégate, *n* = 59. Two clusters represented by gray and white bars.

Microsatellite pairwise *F*_ST_ analyses revealed moderate differentiation between the populations on Aride and Cousine (2011, *F*_ST_ = 0.08, *P *<* *0.001). Subtle differentiation was also detected between all other pairwise comparisons (2011, *F*_ST_ = 0.01–0.05, all *P *<* *0.001) except for between Cousin and Frégate (*P *=* *0.73). The MHC *F*_ST_ analyses also revealed subtle differentiation between Cousine and: Aride (2011, *F*_ST_ = 0.02, *P *=* *0.002), Denis (2011, *F*_ST_ = 0.02, *P *=* *0.005) and Cousin (2011, *F*_ST_ = 0.01, *P *=* *0.03). Pairwise *F*_ST_ are given in Table S2 (Supporting information). A positive correlation was found between microsatellite and MHC *F*_ST_ (*r*_M_ = 0.69, *P *<* *0.001, Fig.3). The structure analysis identified two genetically distinct clusters across the five populations (Fig. S2, Supporting information). When visualized, the islands of Cousin, Denis and Frégate contained a mixture of both clusters, but clear segregation of the clusters was observed in the Aride and Cousine populations (Fig.4).

Estimates of N_e_ varied between methods (Table2), but both methods estimated Cousin to have the largest N_e_ and Cousine to have the smallest N_e_.

**Table 2:** Effective population size estimates for five Seychelles warbler populations using two methods on samples from 2011. Medians are given with credible limits for ONeSAMP and 95% confidence intervals for LDNE

Island	ONeSAMP	LDNE
Cousin	35 (31–42)	68 (59–82)
Aride	24 (21–31)	39 (26–70)
Cousine	23 (21–28)	22 (16–34)
Denis	28 (25–34)	36 (26–61)
Frégate	26 (23–30)	54 (38–85)

## Discussion

The Seychelles warbler, with its translocation history and long-term sampling regime, presents a useful case study of the spatio-temporal impact of conservation translocations on neutral and functional genetic diversity. We found low and temporally stable microsatellite diversity, and low MHC diversity in the source population on Cousin. Our rarefaction models predicted allele retention accurately, and genetic capture (as opposed to subsequent drift) appeared to be the main determinant of translocated population diversity. A small number of rare alleles from neutral markers were lost during translocation, but no statistically significant loss of either neutral or functional diversity was detected in any of the four new populations. Further, diversity in these new populations remained stable over time. Importantly, however, the populations established with a lower number of founders (Aride and Cousine) were central to the low to moderate levels of genetic differentiation observed among populations, indicating that translocations produced subtle changes in allele frequencies, if not in levels of diversity *per se*.

Conservationists have been advised to capture ≥95% of the source population's genetic diversity during translocations to limit any bottleneck effects caused by the translocation process (Weeks *et al.* 2011). Our rarefaction models show that translocations to Denis and Frégate (58 and 59 birds, respectively) captured *ca*. 95% of neutral diversity (87% for diverse loci), but the earlier translocations of 29 birds to Aride and Cousine only captured *ca*. 91% (85% for diverse loci). In line with this, we found a slight, albeit nonsignificant, decrease in both allelic richness and heterozygosity in the Aride and Cousine populations (Fig.2). We would expect a more pronounced effect of loss on allelic richness measures than heterozygosity (Allendorf 1986), but we see no significant difference. A similar study by Taylor and Jamieson (2008) reported little subsequent loss of genetic variation in serial translocations of saddlebacks (*Philesturnus carunculatus*) involving even smaller numbers of founders (lowest *n* = 16), although the source population also had lower initial variation. Our result supports their suggestion that long-term population history (i.e. previous severe bottlenecks) may negate any contemporary bottleneck effects caused by small numbers of founders. This is simply because if all but the commonest alleles are already lost, there is little variation left to lose in subsequent bottlenecks. However, this will only be true for the most extreme cases of genetically depauperate populations (as in the saddlebacks). The Seychelles warbler is severely bottlenecked, and yet we still find some loss of variation during translocations, indicating that care – and suitably high numbers of founders – must be taken to avoid further genetic impoverishment of translocated populations. Furthermore, statistical and biological significance may not concur, as rare alleles can play important roles in evolution (Allendorf 1986). Indeed, another approach to conserving genetic diversity in translocations is to maximize the probability of retaining rare alleles in the founding population (Weiser *et al.* 2012). This would require much larger founder sizes – in our case, our rarefaction curves suggest that to capture 99% of variation (thus capturing the majority of rare alleles), we would have needed to translocate in excess of 130 individuals. However, when planning translocations, conservation managers are inevitably faced with balancing genetic factors against other considerations (namely logistics, expense and restricted source populations), and the best genetic approach may not be possible. Where neutral markers are a good proxy for functional diversity (as our study suggests can be the case in bottlenecked populations), the development of predictive models of neutral diversity loss makes it possible to compare the genetic consequences of different management options prior to translocation (Weiser *et al.* 2013) – a practice that we would encourage wherever possible.

Disentangling the impact of genetic capture, its associated founder effects, and subsequent drift on genetic diversity is generally not possible, requiring virtually complete sampling of a population before, during and, for extended periods, after the translocation event. Complete microsatellite genotypes of all founders on Denis and Frégate enabled us to identify genetic capture as the main determinant of diversity in the translocated populations, and to pinpoint alleles lost specifically through genetic capture. All but two of the alleles that were lost were rare (frequency < 0.01) in the source population, in line with theoretical expectations (Allendorf 1986; Stockwell *et al.*, 1996). The fixation of an allele at locus *Pte24*-CEST on Frégate further demonstrates the stochastic nature of genetic capture. This locus became fixed in the largest founding population, whilst remaining biallelic across three other translocations of smaller founder numbers. Our data demonstrate that the loss of rare alleles in the founding populations is due to incomplete genetic capture. Additionally, there is little evidence for significant changes in diversity over time, suggesting that drift subsequent to the translocations has had little effect on these populations. This may partly be explained by the rapid population growth observed in the translocated populations, which will have limited the effects of drift (Nei *et al.* 1975).

The rarefaction models for MHC diversity suggested that *ca*. 20–25 Seychelles warblers would be required to capture all the known source MHC variation, which was achieved in all translocations. MHC copy number variation may exist in these populations, but it is difficult to separate from the variance in number of alleles shared across duplicated loci (e.g. Eimes *et al.* 2011). It may be therefore logical to translocate individuals with the largest number of different alleles, irrespective of whether this was the result of copy number variation or across-loci heterozygosity, as this would maximize the MHC variation in the founding population.

The Seychelles warbler possesses a depauperate MHC diversity compared with other *Acrocephalus* species, with only ten class I alleles observed (Hansson & Richardson 2005) and no class II variation detected (Hutchings 2009). Pathogen-mediated balancing selection is believed to maintain MHC diversity in large populations (Doherty & Zinkernagel 1975; Slade & McCallum 1992; Spurgin & Richardson 2010). However, with decreasing population size, the effects of drift are more severe, and selection needs to be stronger to maintain diversity (Kimura 1983). Studies have shown that neutral processes outweigh selection in shaping MHC diversity in small, bottlenecked and/or isolated populations (e.g. Seddon & Ellegren 2004; Miller *et al.* 2010; Agudo *et al.* 2011; Sutton *et al.* 2011), although instances of selection acting to maintain MHC diversity have been documented (e.g. Aguilar *et al.* 2004; Oliver & Piertney 2012). Here, we find that the pattern of microsatellite and MHC differentiation is highly positively correlated (Fig.3), suggesting that any MHC-based selection that occurs in the Seychelles warbler (Richardson *et al.* 2005; Brouwer *et al.* 2010) is not strong enough to override the effect of neutral processes during translocations. Instead, our data support the conclusion that demographic processes shape both neutral and functional diversity in a similar way. In our case, it seems that the stochastic process of genetic capture of small founder numbers has been the main driver of differentiation between translocated Seychelles warbler populations (Fig.4). Although our analysis clearly suggests k = 2 genetic clusters across the populations, it can be interpreted as three ‘groupings’: the heterogeneous populations of Cousin, Denis and Frégate forming one group and Aride and Cousine each forming another, with the latter two diverging in opposite directions to one another due to their smaller numbers of founders.

Overall, there appears less variation in the MHC than observed at the microsatellites. Admittedly, these particularly variable microsatellites were originally selected from a larger panel of markers to resolve parentage, which may explain their sensitivity in detecting population differentiation, where MHC was less suited. An obvious but key point is that the observable impact of translocations on genetic diversity is wholly dependent on the variability of the loci used in the study. Careful choice of good, highly polymorphic markers is therefore important to enable higher resolution in studies investigating genetic variation loss.

Defining a ‘successful’ translocation is difficult and can lead to misinterpretation regarding long-term possibility of failure and, potentially, to inadequate future conservation effort (Griffith *et al.* 1989; Fischer & Lindenmayer 2000). Two biologically relevant aspects on which success can be judged are persistence and resilience (Fischer & Lindenmayer 2000). Many avian translocation studies report high mortality during/immediately following release and often complete failure of populations to become established (e.g. Brekke *et al.* 2011; Jamieson 2011; White *et al.* 2012). The Seychelles warbler programme is therefore unusual, with no mortality occurring during any of the four translocations (Komdeur 1994; Richardson *et al.* 2006; Wright & Richardson 2012). No loss of variation is observed subsequently in the translocated populations (after the loss due to the initial genetic capture). This indicates that survival is high, that reproductive representation of founders is balanced and that the rapid population growth observed during establishment has limited the severity of the founder bottleneck. Current census population estimates for each island are Cousin = 320, Aride = 1850, Cousine = 210, Denis = 300 and Frégate = 80. The populations on Cousin, Aride and Cousine are at carrying capacity (DSR, pers. obs.), with differences in the area and quality of habitat on the different islands responsible for the variation in island capacity. Denis and Frégate are also still in the early stages of post-translocation population growth. The translocations can therefore be considered extremely successful in the short-medium term. The long-term genetic resilience of a population will depend on the functional variation present. Other bottlenecked species, such as southern elephant seals (*Mirounga leonina*), Chatham Island black robins (*Petroica traversi*), cheetah (*Acinonyx jubatus*) and falcons (*Falco* spp.), survive despite extremely low MHC variability (Slade 1992; Miller & Lambert 2004; Castro-Prieto *et al.* 2011; Gangoso *et al.* 2012). Decreased pathogen exposure within such populations may partly explain their apparent viability (Slade 1992; Miller & Lambert 2004), and depauperate parasite loads are found in the Seychelles warbler (Hutchings 2009). However, whether the remaining MHC diversity provides adequate resilience against any novel pathogens that may enter these populations in the future is unknown.

The variability in effective population size (N_e_) estimates between the methods means caution should be exercised with interpretations. Both the severity and duration of bottleneck events will affect estimates of N_e_ (Nei *et al.* 1975; Frankham 1995). Although the bottlenecks were relatively severe (29 and 58 of 59), all founders were sourced from an already bottlenecked population (Spurgin *et al*. in review). Further, each population has experienced rapid growth over many generations (with the exception of Frégate, only established at the end of 2011), which would limit further reduction in N_e_. The general scale and order of N_e_ estimates across populations therefore appear logical in relation to known history, as well as founder and census population sizes. Our results show that while the translocations have clearly been very successful in massively expanding the overall Seychelles warbler population and range without significant extra loss of diversity, most of the estimates of N_e_ for our populations fall below any recommended minimum viable sizes (50–5000, Lande 1995; Franklin & Frankham 1998). This and the low levels of diversity at both neutral and functional loci mean that concerns regarding the evolutionary potential of this species still cannot be discounted. Given these results and the evidence that some genetic divergence exists, assisted gene flow between translocated populations may be required in the future to maintain all the Seychelles warblers as one large, undifferentiated and hence more viable population. The general environmental conditions are similar between islands (indeed host islands were selected based on their similarity to Cousin); but we cannot fully discount the possibility of different selection pressures across populations due to factors we have not been able to assess. It may therefore be logical to check for adaptive differences between populations before undertaking assisted gene flow as this could cause outbreeding depression, an important consideration for translocation projects in general (Edmands 2007).

## Conclusion

The demographic history of the Seychelles warbler is typical of many endangered species (Hudson *et al.* 2000; Robertson *et al.* 2009; Grueber & Jamieson 2011; Bristol *et al.* 2013), and our results should therefore be of general applicability. Questions have been raised about inferring functional variation based solely on any one given locus, that is the MHC (Acevedo-Whitehouse & Cunningham 2006; Radwan *et al.* 2010). Future work on other important immune genes, such as toll-like receptors (e.g. Grueber *et al.* 2013), individual variation in gene copy number (e.g. Eimes *et al.* 2011) and broader genomic studies (Angeloni *et al.* 2012), will help our understanding of how and why genetic variation influences population persistence. What role candidate genes vs. genomic approaches will play in conservation programmes will also undoubtedly be an important avenue of research. However, conservation biology is a crisis discipline (Soulé 1985) and requires efficient, evidence-based decision-making. From a practical perspective, in the case of the Seychelles warbler, investigating MHC variation as an example of functional genetic diversity provided no extra information (above and beyond that of the microsatellite data) to help estimate the numbers of founders required. Evidence is now accumulating for drift outweighing selection in small populations and that maintaining maximal genetic diversity is vital in species conservation. Based on this, we suggest that in bottlenecked, isolated species, such as the Seychelles warbler, genetic decisions on the number of founders to use in translocations could be made most efficiently and cost-effectively based on neutral diversity measures. However, this depends on the demographic history of the organism. It may be that this is an acceptable course of action only in bottlenecked, genetically depauperate populations, where drift has largely overridden selection. In our study, a handful (e.g. five) of the most polymorphic microsatellite loci would have allowed us to accurately determine the required number of founders to be translocated and to monitor the genetic diversity of the resulting populations. Further, using a suite of markers means that decisions are not made on a single functional region at the potential expense of other important regions (Radwan *et al.* 2010). Lastly, as next-generation sequencing technologies become cheaper and hence more accessible to conservation programmes, it should be possible to provide conservation managers with increasingly accurate information on genome-wide variation within and between populations to aid vital evidence-based decision-making (Angeloni *et al.* 2012). The results presented here add to the growing evidence base on the impact and use of translocations as an important conservation tool and will, we hope, help inform translocation practices and the conservation of other endangered species.

## Notes

D.S.R. and D.J.W. designed the study. The long-term fieldwork and translocations were managed by D.S.R. and J.K. The molecular analyses were undertaken in the laboratories of D.S.R., J.K. and T.B. D.J.W. and L.G.S. analysed the data. D.J.W., L.G.S. and D.S.R. drafted the manuscript, with input from N.J.C., T.B. and J.K. All authors edited and agreed to the manuscript.

## Data accessibility

Individual microsatellite and MHC genotypes with catch year and island have been deposited in the DRYAD data repository (doi:10.5061/dryad.596k1).

## Supporting Information

Fig S1 Map of the inner Seychelles archipelago with location, date and number of founding individuals for the four Seychelles warbler translocations.

Fig S2 Graph generated by STRUCTURE HARVESTER (Earl & vonHoldt 2012), displaying the change in ΔK against number of clusters (K) calculated following the method of Evanno *et al*. (2005), highlighting that K = 2 is the most likely number of genetic clusters across five island populations of Seychelles warbler.

Table S1 Null allele estimates for each of 30 microsatellite loci across each catch-year sample period for five island populations of Seychelles warblers where CN=Cousin, AR=Aride, CE=Cousine, DS=Denis and FR=Frégate. Estimates >0.1 highlighted in bold type.

Table S2 Pairwise F_ST_ between each population sample across five island populations of Seychelles warblers. Microsatellites (lower) and major histocompatibility complex (upper) data.
