# Supplementary material for: The impact of translocations on neutral and functional genetic diversity within and among populations of the Seychelles warbler
Source: Mol Ecol. 2014 Apr 18;23(9):2165–77. doi: 10.1111/mec.12740 (PMC4237152; doi:10.1111/mec.12740)

Supplementary figure 1: Map of the inner Seychelles archipelago with location, date and number of founding individuals for the four Seychelles warbler translocations. The source population Cousin is highlighted in bold. Longitude and latitude for each island is provided in the materials and methods of the article.

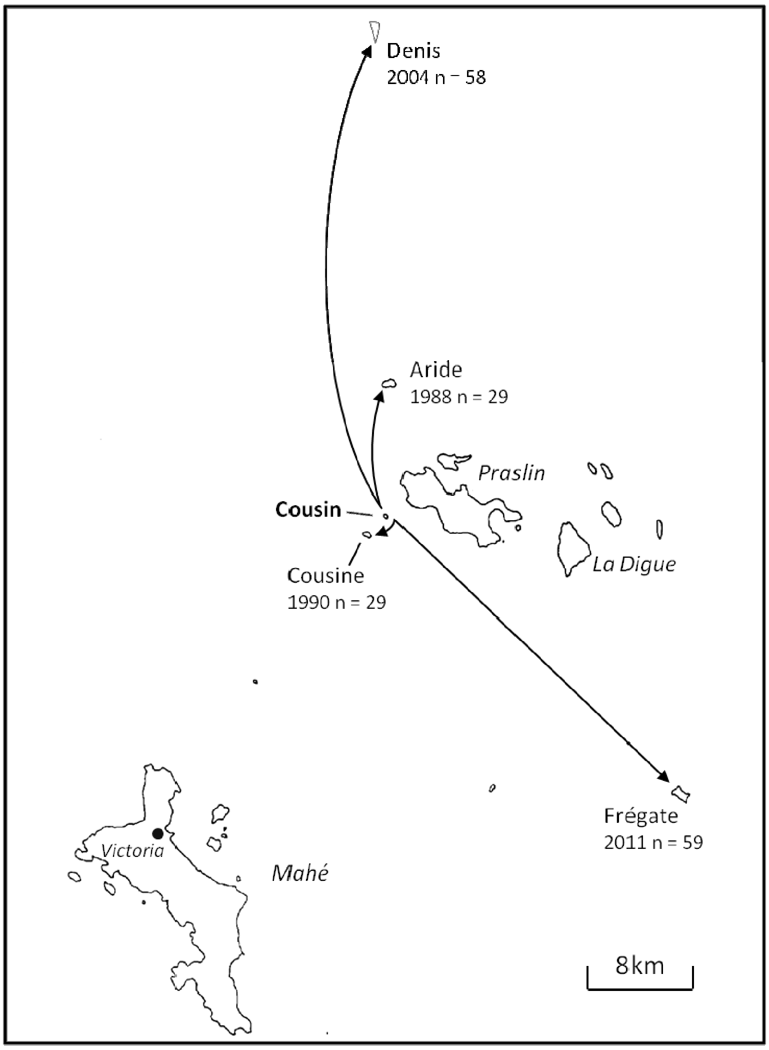

Supplement: Fig S1 — Map of the inner Seychelles archipelago with location, date and number of founding individuals for the four Seychelles warbler translocations. [file mec0023-2165-SD1.pdf]
