# Supplementary material for: The impact of translocations on neutral and functional genetic diversity within and among populations of the Seychelles warbler
Source: Mol Ecol. 2014 Apr 18;23(9):2165–77. doi: 10.1111/mec.12740 (PMC4237152; doi:10.1111/mec.12740)

Supporting Figure 2: Graph generated by STRUCTURE HARVESTER (Earl and vonHoldt, 2012), displaying the change in ΔK against number of clusters (K) calculated following the method of Evanno *et al*. (2005), highlighting that K = 2 is the most likely number of genetic clusters across five island populations of Seychelles warbler.

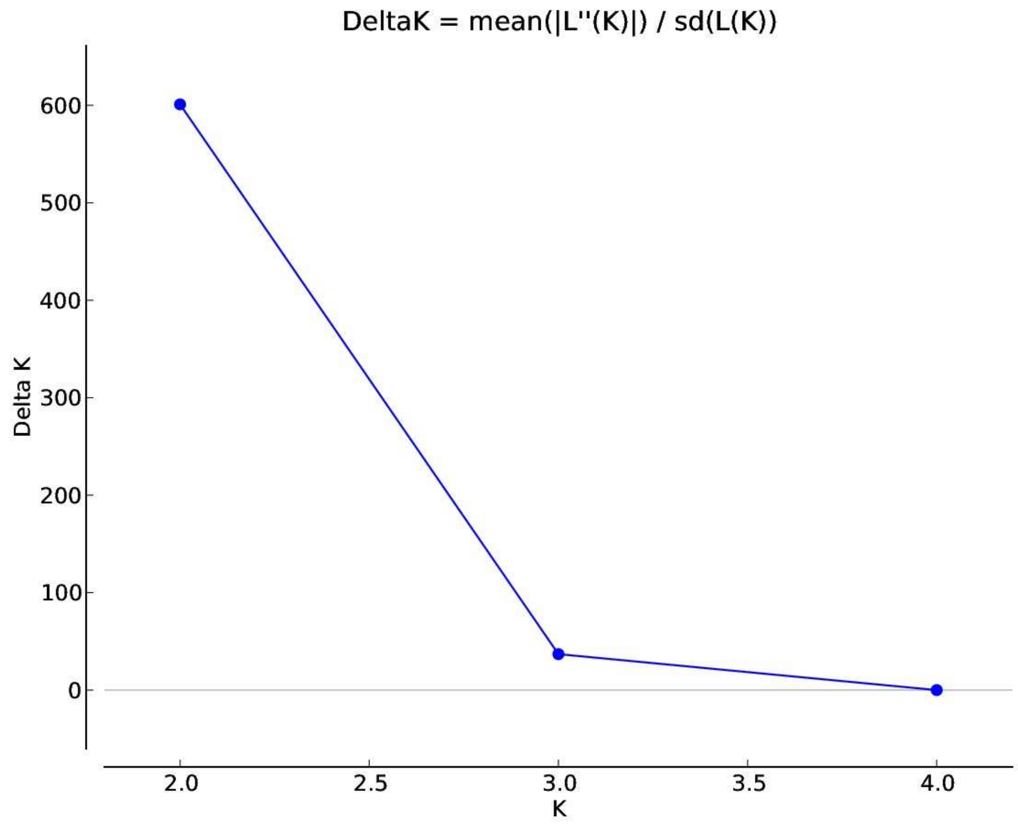

Supplement: Fig S2 — Graph generated by STRUCTURE HARVESTER (Earl & vonHoldt 2012), displaying the change in ΔK against number of clusters (K) calculated following the method of Evanno et al. (2005), highlighting that K = 2 is the most likely number of genetic clusters across five island populations of Seychelles warbler. [file mec0023-2165-SD2.pdf]
