# Supplementary material for: The impact of translocations on neutral and functional genetic diversity within and among populations of the Seychelles warbler
Source: Mol Ecol. 2014 Apr 18;23(9):2165–77. doi: 10.1111/mec.12740 (PMC4237152; doi:10.1111/mec.12740)
Supplement: Table S1 — Null allele estimates for each of 30 microsatellite loci across each catch-year sample period for five island populations of Seychelles warblers where CN=Cousin, AR=Aride, CE=Cousine, DS=Denis and FR=Frégate. Estimates >0.1 highlighted in bold type. [file mec0023-2165-SD3.pdf]

Supplementary table 1: Null allele estimates for each of 30 microsatellite loci across each catch-year sample period for five island populations of Seychelles warblers where CN = Cousin, AR = Aride, CE = Cousine, DS = Denis and FR = Frégate. Estimates > 0.1 highlighted in bold type.

**Locus**	**CN 1993**	**CN 2005**	**CN 2011**	**AR 1993**	**AR 2005**	**AR 2011**	**CE 1997**	**CE 2005**	**CE 2011**	**DS 2004**	**DS 2011**	**FR 2011**
Ase10	-0.04	-0.07	-0.05	-0.11	0.02	**0.24**	0.00	-0.08	0.04	-0.06	-0.09	-0.11
Ase13	-0.08	-0.03	-0.05	-0.12	0.01	-0.07	0.08	-0.06	0.03	0.06	-0.01	0.01
Ase18	-0.04	0.01	0.00	-0.04	-0.09	0.00	0.01	-0.04	-0.02	0.00	-0.09	-0.06
Ase25	-0.03	0.04	-0.01	-0.06	-0.07	-0.07	-0.04	-0.08	-0.08	-0.01	0.01	-0.03
Ase27	0.02	-0.02	-0.03	-0.03	-0.02	0.09	-0.11	**0.15**	-0.01	-0.05	0.01	-0.04
Ase35	0.01	0.07	0.00	-0.02	-0.06	0.02	-0.01	0.07	**0.17**	-0.04	0.07	0.00
Ase37	0.02	0.03	-0.02	-0.06	-0.05	0.10	0.02	-0.02	0.01	0.04	-0.04	-0.04
Ase4	-0.08	0.01	-0.07	**0.40**	-0.09	-0.07	0.10	-0.01	**0.16**	-0.03	0.01	0.02
Ase42	-0.11	-0.04	0.02	-0.12	-0.06	0.07	-0.05	**0.11**	0.07	-0.05	0.06	-0.02
Ase48	-0.02	-0.01	-0.10	-0.11	-0.05	-0.14	-0.03	-0.11	0.02	-0.03	0.06	-0.02
Ase56	-0.03	0.05	0.01	-0.08	-0.06	-0.05	-0.06	-0.04	-0.04	0.05	-0.01	**0.14**
Ase58	-0.02	0.02	-0.03	0.01	-0.10	-0.11	-0.05	0.06	-0.04	-0.04	0.00	-0.04
Ase6	-0.02	0.02	0.01	0.00	-0.04	-0.03	-0.04	-0.03	-0.07	-0.03	-0.09	0.04
Ase9	-0.06	-0.01	0.04	0.03	-0.13	-0.10	-0.17	0.04	-0.07	0.03	0.00	-0.03
Ase11	-0.09	0.00	0.01	-0.04	-0.12	-0.09	**0.20**	-0.03	0.07	-0.02	-0.08	**0.11**
Ase16	0.01	-0.01	0.02	-0.04	-0.06	0.01	0.05	-0.03	-0.07	0.01	-0.02	-0.05
Ase19	0.07	0.00	-0.04	-0.06	-0.07	**0.17**	0.08	-0.02	-0.05	0.01	0.08	0.00
Ase22	-0.13	0.09	-0.01	**0.11**	-0.03	-0.08	0.09	-0.08	0.09	0.01	**0.11**	0.09
Ase3	-0.12	-0.02	0.03	**0.10**	**0.21**	**0.14**	-0.02	-0.02	0.04	-0.01	**0.15**	0.02
Ase38	-0.04	0.00	-0.02	-0.23	0.00	-0.08	-0.16	0.03	0.04	-0.03	-0.12	**0.13**
Ase53	0.00	-0.04	0.06	**0.11**	-0.09	0.00	-0.06	0.05	0.06	0.06	-0.04	-0.03
Ase55-CEST	-0.03	0.06	-0.01	-0.03	-0.20	0.03	-0.03	0.05	-0.01	0.03	-0.07	-0.06
Ase61	0.05	0.06	0.04	-0.08	-0.01	**0.11**	0.00	-0.14	-0.12	0.00	-0.03	0.02
Ase64	0.03	-0.01	-0.03	0.02	-0.06	0.04	0.10	-0.07	-0.05	0.04	-0.12	-0.03
Ase7	-0.04	0.08	-0.05	-0.08	0.07	-0.04	0.00	0.01	0.00	0.02	-0.13	-0.07
Calex-08-Gga	**0.13**	0.00	-0.02	-0.11	-0.16	**0.11**	0.00	0.00	0.00	-0.04	0.03	-0.03
Cuμ4-Gga5	0.01	0.01	-0.09	-0.15	-0.02	-0.04	-0.12	-0.02	-0.07	**0.12**	0.02	-0.08
Pdoμ6	0.03	0.02	0.01	0.02	-0.06	0.02	0.00	0.03	-0.13	0.03	0.07	0.02
PmaTGAn42	0.03	0.00	-0.06	**0.31**	-0.07	0.09	0.00	0.04	-0.11	-0.07	-0.06	**0.12**
Pte24-CEST	0.00	0.00	0.00	0.00	0.00	-0.01	0.00	0.00	0.00	0.00	0.00	0.00
