# Supplementary material for: The impact of translocations on neutral and functional genetic diversity within and among populations of the Seychelles warbler
Source: Mol Ecol. 2014 Apr 18;23(9):2165–77. doi: 10.1111/mec.12740 (PMC4237152; doi:10.1111/mec.12740)
Supplement: Table S2 — Pairwise FST between each population sample across five island populations of Seychelles warblers. Microsatellites (lower) and major histocompatibility complex (upper) data. [file mec0023-2165-SD4.pdf]

Supplementary table 2: Pair-wise F_{ST} between each population sample across five island populations of Seychelles warblers. Microsatellites (lower) and major histocompatibility complex (upper) data.

	**CN 1993**	**CN 2005**	**CN 2011**	**AR 1993**	**AR 2005**	**AR 2011**	**CE 1997**	**CE 2005**	**CE 2011**	**DS 2004**	**DS 2011**	**FR 2011**
**CN 1993**		0.000	0.000	0.005	0.011	0.006	0.001	0.003	0.004	0.000	0.004	0.000
**CN 2005**	0.002		0.000	0.003	0.008	0.004	0.000	0.004	0.006	0.000	0.001	0.000
**CN 2011**	0.006	0.003		0.000	0.003	0.000	0.000	0.005	0.009	0.000	0.000	0.000
**AR 1993**	0.012	0.019	0.023		0.000	0.000	0.000	0.011	0.021	0.000	0.000	0.000
**AR 2005**	0.034	0.037	0.040	0.006		0.000	0.000	0.019	0.030	0.005	0.000	0.004
**AR 2011**	0.033	0.034	0.039	0.006	0.017		0.000	0.014	0.023	0.001	0.000	0.000
**CE 1997**	0.026	0.022	0.023	0.037	0.064	0.056		0.004	0.010	0.000	0.000	0.000
**CE 2005**	0.067	0.049	0.048	0.072	0.092	0.086	0.007		0.000	0.008	0.011	0.005
**CE 2011**	0.045	0.032	0.032	0.058	0.085	0.084	0.006	0.000		0.011	0.018	0.008
**DS 2004**	0.000	0.005	0.008	0.011	0.033	0.030	0.026	0.060	0.042		0.000	0.000
**DS 2011**	0.008	0.017	0.013	0.016	0.038	0.040	0.038	0.071	0.049	0.002		0.000
**FR 2011**	0.005	0.003	0.000	0.024	0.040	0.040	0.020	0.046	0.029	0.006	0.011	
